# Translation of *in vitro*-transcribed RNA therapeutics

**DOI:** 10.3389/fmolb.2023.1128067

**Published:** 2023-02-08

**Authors:** Tobias von der Haar, Thomas E. Mulroney, Fabio Hedayioglu, Sathishkumar Kurusamy, Maria Rust, Kathryn S. Lilley, James E. Thaventhiran, Anne E. Willis, C. Mark Smales

**Affiliations:** ^1^ School of Biosciences, Division of Natural Sciences, University of Kent, Canterbury, United Kingdom; ^2^ MRC Toxicology Unit, Gleeson Building, University of Cambridge, Cambridge, United Kingdom; ^3^ Department of Biochemistry, University of Cambridge, Cambridge, United Kingdom

**Keywords:** RNA therapeutic, translation, protein synthesis, translational control, RNA vaccines

## Abstract

*In vitro* transcribed, modified messenger RNAs (IVTmRNAs) have been used to vaccinate billions of individuals against the SARS-CoV-2 virus, and are currently being developed for many additional therapeutic applications. IVTmRNAs must be translated into proteins with therapeutic activity by the same cellular machinery that also translates native endogenous transcripts. However, different genesis pathways and routes of entry into target cells as well as the presence of modified nucleotides mean that the way in which IVTmRNAs engage with the translational machinery, and the efficiency with which they are being translated, differs from native mRNAs. This review summarises our current knowledge of commonalities and differences in translation between IVTmRNAs and cellular mRNAs, which is key for the development of future design strategies that can generate IVTmRNAs with improved activity in therapeutic applications.

## 1 Introduction

The notion that *in vitro* transcribed modified mRNAs (IVTmRNAs) could be used for therapeutic purposes originates from experiments conducted in the 1990s. These early experiments demonstrated that the injection of pure, *in vitro* transcribed mRNA into mouse muscle resulted in the detectable production of the encoded protein (Wolff et al., 1990). Early delivery mechanisms utilised liposomes (Zhang et al., 2022) which were subsequently superseded by the development of lipid nanoparticle (LNP) formulations which enhanced tissue uptake of the RNAs while at the same time protecting them efficiently from nuclease digest (Hou et al., 2021). Naked (non-protein bound) IVTmRNAs are prone to elicit immune responses, which could be controlled through the development of chemical modifications that suppress the immunogenicity of the RNA molecule (Karikó et al., 2005). These were key developments in the journey into applications as vaccines, which ultimately led to the rapid development of the COVID-19 vaccines, many other vaccines currently in clinical development, and a growing array of additional applications beyond vaccines (Damase et al., 2021; Zhang et al., 2022).

IVTmRNA therapeutics are translated into proteins by the cellular protein synthesis machinery. It may be assumed that the translation of such RNAs uses the same or similar pathways as natural endogenous *in vivo* transcribed RNAs. While this is likely true in the most general terms, there is considerable scope for variation in how the cell translates such RNAs. This topic is little discussed, and variations in the process of protein synthesis between natural endogenous transcripts and IVTmRNAs are in general not well understood. Patents on the existing COVID-19 vaccines as well as the documented thought processes that led the to the approval of IVTmRNAs for use in the clinic^1^ strongly focus on formulation and on the immune responses elicited by both the RNA and the encoded proteins, whereas little attention is paid to the actual processes by which the IVTmRNAs are translated.

At the time of writing, billions of IVTmRNA doses have been administered. Given the widespread use, a better understanding of the mechanisms of action of coding therapeutic RNAs, including the process of translation into protein, is key. Moreover, current sequence design approaches are based on those commonly used for recombinant DNA constructs (see below), and better understanding of IVTmRNA-specific translation mechanisms is required to generate more efficient sequence design approaches. With these aims in mind, this review summarises the current literature and understanding of the mechanism of protein synthesis from IVTmRNAs and its relation to protein synthesis on natural transcripts.

## 2 Cytoplasmic entry and engagement with the translational machinery of mRNAs

**FIGURE 1:**
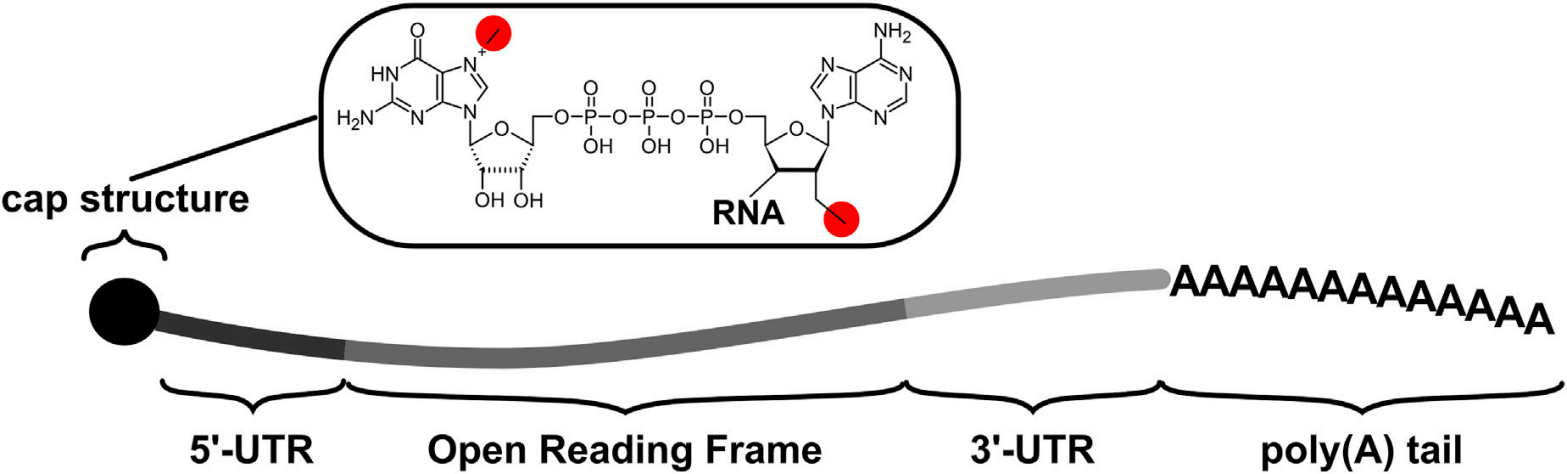
The principal components of native transcripts as well as IVTmRNAs comprise untranslated regions (UTRs) which flank the protein-coding Open Reading Frame or ORF. Cap structures are introduced co-transcriptionally *in vivo* and may be introduced co- or post-transcriptionally *in vitro*. The poly(A) tail is added post-transcriptionally *in vivo*, and may be added as part of the transcribed sequence or post-transcriptionally *in vitro*. The chemical detail illustrates the cap1 structure which is characterized by the two methyl group highlighted in red (alternative cap structures differ in the number and locations of the methyl groups).

All eukaryotic mRNAs, whether mature endogenous transcripts or IVTmRNAs, consist of a number of non-coding elements alongside the protein coding open reading frame (ORF, Figure 1). The non-coding regions usually include a 5′ 7-methyl-GTP cap and a 5′ untranslated region (UTR) of variable length in front of the ORF, and a 3’ UTR (again of variable length) following the ORF. Transcripts end in a poly(A) tail structure, which have typical initial lengths of 200–250 nucleotides in humans (Eckmann et al., 2011) but are typically 130 nucleotides in IVTmRNAs if they are encoded on the *in vitro* transcription template. All these UTR elements play key roles in recruiting ribosomes and translation factors to the RNA.

**FIGURE 2:**
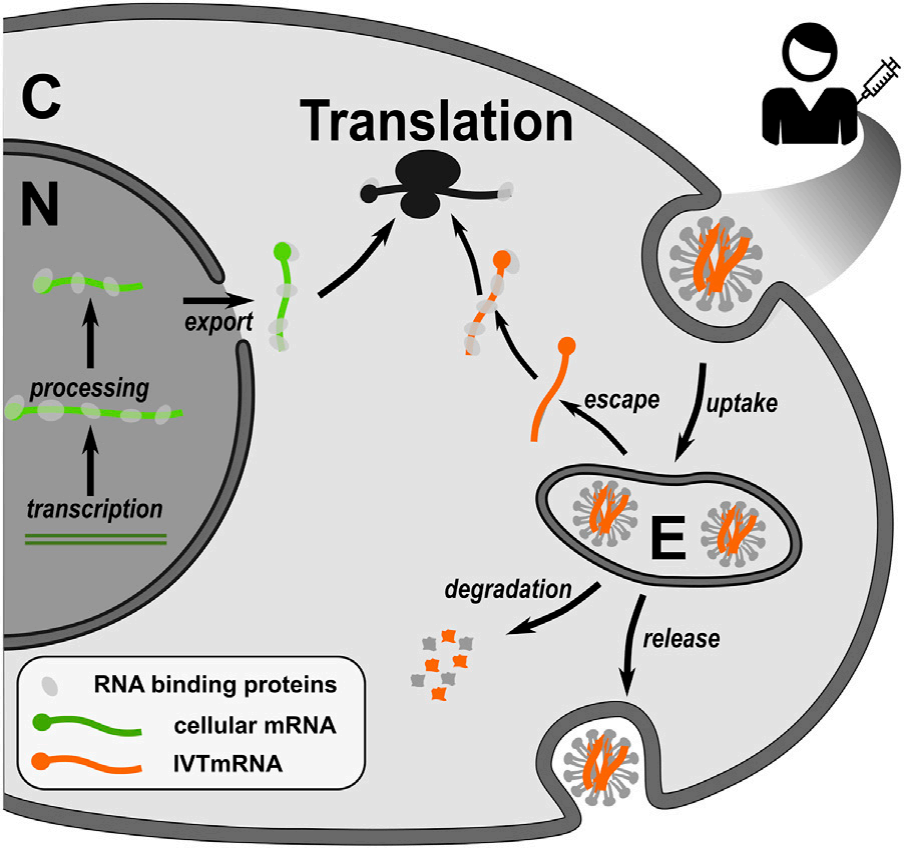
Native endogenous transcripts and IVTmRNAs have different genesis pathways which determine differences in translational efficiency. Native transcripts engage with the translational machinery following nuclear processing and export, in a protein-bound mRNP state where mRNP composition is controlled by the preceding processing steps. IVTmRNAs enter cells through the endosomal pathway, from which they escape inefficiently. In how far IVTmRNAs are able to form functional mRNPs is unknown. C, cytoplasm, N, nucleus, E, endosome.

Whilst such elements are common between IVTmRNAs and endogenous mRNAs, their effect on translation can differ between natural transcripts and IVTmRNAs due to their differing routes of entry into the cytoplasm and differing modes of engagement with the translational machinery (Figure 2). A particular hallmark of the natural pathway is that the processes of mRNA maturation and export deposit combinations of protein-based markers and RNA nucleotide modifications that record the transcripts’ genesis pathway. Such protein and RNA “marks” have the potential to affect translation in multiple ways. These marks are entirely absent from IVTmRNAs when they first engage with the translational apparatus, which can thus be described as a “naked” mRNA.

### 2.1 The native RNA pathway

Native mRNAs engage the translational machinery following nuclear transcription, processing and export to the cytoplasm. During this journey, transcripts do not exist as naked RNAs, instead, they associate with RNA binding proteins to form messenger Ribonucleoproteins (mRNPs). According to the current view in the field, mRNPs change composition as they progress through the different steps of a transcript’s life cycle, in a manner that involves coordinated handover events between the different protein complexes (Pichon et al., 2012). An important aspect of this concept is that the distinct protein components of the mRNP record the life history of a transcript, allowing events early on in a transcript’s history to affect stability, localisation, and translational activity later on. Relevant concepts have been reviewed in depth (Singh et al., 2015; Björk and Wieslander, 2017; Gehring et al., 2017; Khong and Parker, 2020; Zarnack et al., 2020). Here, we focus on steps leading to the first engagement with the translational machinery which are most distinct for endogenous native mRNAs compared to IVTmRNAs.

Hallmarks of nuclear mRNPs following transcription, splicing and export include a dimeric nuclear cap-binding complex (CBC) on the mRNA cap structure. On spliced mRNAs, an additional hallmark is the presence of exon junction complexes (EJCs) which are deposited over exon:exon junctions during splicing and which signal the location of such sites to the translational machinery and the cellular mRNA surveillance pathways. The first or “pioneer” round of translation is thought to occur on mRNPs in this state (Ishigaki et al., 2001). During the pioneer round EJCs are removed from the transcript by the translating ribosome. Because on native mRNAs introns are usually restricted to 5′-UTRs and the open reading frame, but are generally excluded from 3′-UTRs, ribosomes normally terminate on transcripts where all EJCs have been removed. Termination upstream of a remaining EJC thus flags the presence of a premature termination codon and elicits Non-sense Mediated Decay (NMD), one of the cellular mRNA surveillance pathways. Connections between the pioneer round of translation and other quality control pathways have been demonstrated but are less well understood than for NMD. For example, the CBC was recently reported to interact with the RNA component of the signal recognition particle, thereby supporting appropriate targeting of signal sequence-containing proteins to the ER (Park et al., 2021). CBC-dependent translation was also reported to support some forms of antigen presentation, for example on MHC-I (Weinstein-Marom et al., 2019) and on class I HLA (Uchihara et al., 2022).

**FIGURE 3:**
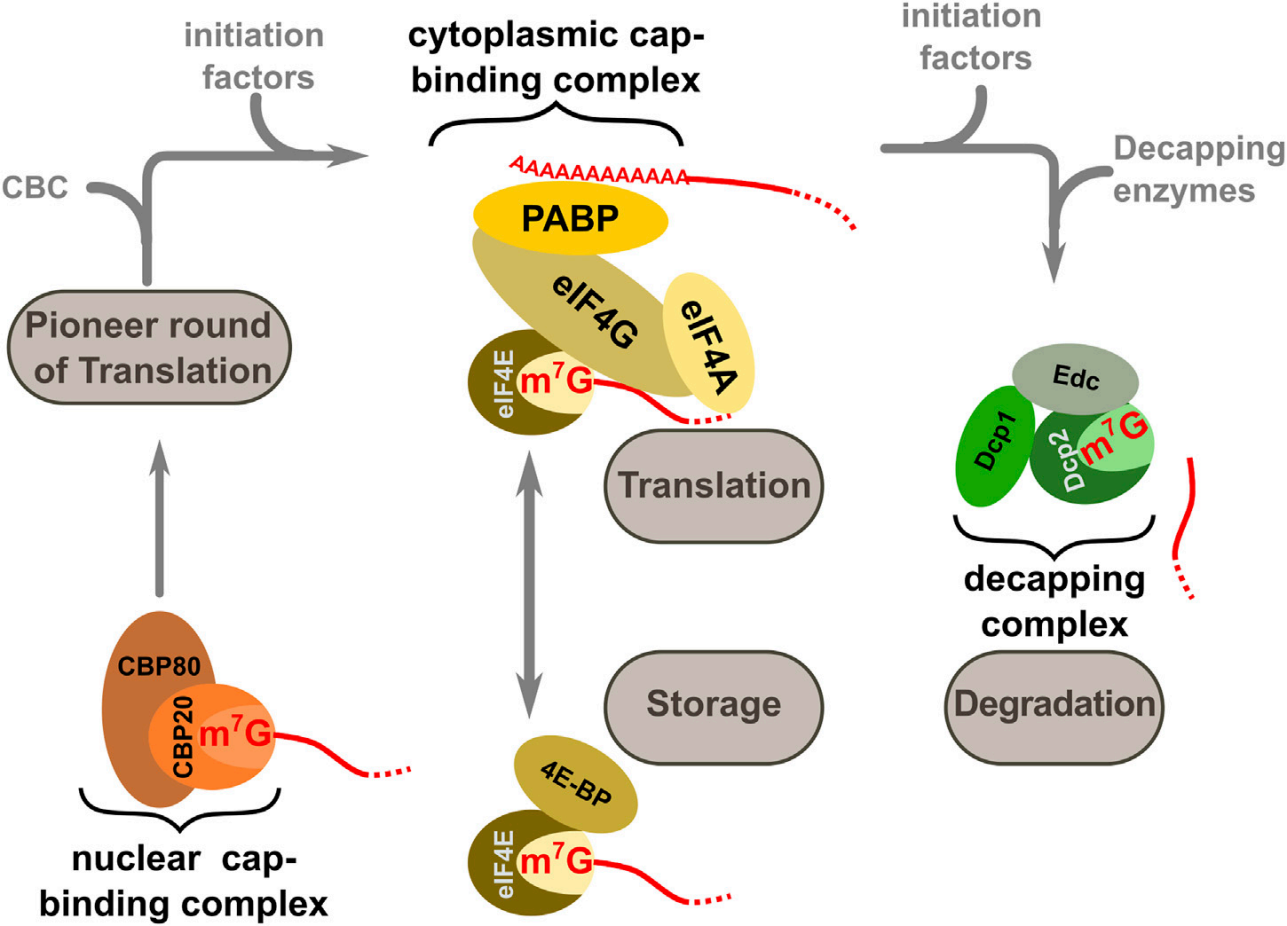
Different cap-binding complexes mediate entry into and exit from the translational state of mRNAs in the cell. The nuclear cap binding complex CBC (consisting of CBP20 and CBP80) is present during nuclear processing events. Following export, this complex is exchanged against a cytoplasmic cap-binding complex comprising members of the eIF4 group of translation factors and the poly(A) binding protein PAB (this complex is collectively termed eIF4F). The productive eIF4F complex can be temporarily disrupted by the 4E binding proteins or 4E-BPs and in this inactive form transcripts may be stored in specific cellular structures such a P-bodies. At the end of the translationally active phase the eIF4F complex is disassembled and replaced by the decapping factors.

Over the lifetime of natural transcripts, association between the cap-structure and different cap-binding complexes and enzymes is one of the important signals that accompany entry into and exit from the translationally active state (Figure 3). Following the pioneer round of translation, CBC is replaced by the cytoplasmic cap-binding protein or eukaryotic initiation factor (eIF) 4E, which is part of the eIF4F complex that additionally contains eIF4A, eIF4G, and the poly(A) tail binding protein PAB. This complex forms a central hub for active translation and ribosome recruitment, *via* interactions with additional translation initiation factors. The active translational state can be interrupted by several eIF4E binding proteins which disrupt eIF4E:eIF4G contacts (Lin et al., 1994; Clemens, 2001), and translational activity can be modulated by the formation of complexes containing different eIF4E and eIF4G isoforms (Landon et al., 2014; Ho et al., 2016). Following a period of translation which coincides with gradual shortening of the poly(A) tail, eIF4E is eventually replaced by decapping enzyme-containing complexes which initiate the mRNA decay process (Fenger- Grøn et al.,. 2005). The CBC-eIF4E handover has been reported to be catalysed by components of the nuclear export machinery (Sato and Maquat, 2009). Moreover, there is an emerging role for RGG-motif containing proteins, which also begin to interact with mRNPs around the time of mRNA export (Poornima et al., 2021), in both CBC-eIF4E and eIF4E-decapping complex handover events (Chowdhury and Jin, 2022).

### 2.2 The IVTmRNA pathway

All RNA therapeutics currently used in the clinic are transcribed *in vitro* from tilizing DNA templates in which the 5′-UTR, ORF, 3′-UTR and poly(A) tail are placed downstream of a T7 promoter sequence (Whitley et al., 2022). A number of strategies are available for introducing the 5′-cap structure and poly(A) tail. For capping, post-transcriptional enzymatic strategies are available (usually based on the capping enzymes from vaccinia virus), as well as non-enzymatic co-transcriptional strategies (Muttach et al., 2017). Current vaccines predominantly use the co-transcriptional route to introduce double methylated “cap1” structures (Figure 1), and achieve capping efficiencies of 90%–95%. For polyadenylation, a poly(A) tail can be encoded on the transcription template but poly(A) tail length is then restricted to around 130 nucleotides due to DNA synthesis constraints. A strategy based on “segmented” poly A tails has been proposed in order to avoid problems with synthesis and stability of template-encoded pure poly(A) tails (Trepotec et al., 2019). Alternatively, poly(A) tails can be introduced enzymatically following *in vitro* transcription which allows generation of longer tails, although this introduces tail length heterogeneity.

Transcribed and capped RNA is purified away from the DNA template and reaction impurities, before being encapsulated in lipid nanoparticles (LNPs, Mukai et al., 2022). LNPs typically consist of mixtures of ionizable lipids, modified polyethylene glycol moieties, and varying helper lipids that form micellar structures around a mixture of the nucleic acid cargo and cholesterol. The different lipids each have defined roles, and LNP composition changes in controlled ways upon injection into patient tissues: for example, the PEG derivatives stabilize LNPs in blood, but need to be shed with controlled rates to enable efficient cellular uptake, whereas the ionizable lipids attract varying surface charges during passage through the endosome thereby promoting endosomal escape at specific times following endocytosis. It has been noted that significant amounts of impurities in the form of truncated IVTmRNAs can still be present in these purified formulations^1^, the impact of which is currently poorly understood.

The fate of individual lipid nanoparticles and their RNA cargo has been determined using a mixture of approaches, relying primarily on cultured cells and animal models. Transfection efficiency varies between cultured cells and intact tissues (Paunovska et al., 2018) and between the same tissue in different animal species (Hatit et al., 2022). Results obtained with cultured cells may thus not be directly transferable to events in tissues of vaccine recipients. However, data from these studies have revealed the principal mechanisms by which IVTmRNAs enter cells and engage with the translational machinery.

The local LNP concentration around the injection site stabilises within the first few hours post-injection (Hassett et al., 2019), during which time the LNPs diffuse into the local muscle and drain into adjacent lymph nodes (Lindsay et al., 2019). A small proportion of LNPs also spreads systemically and reaches organs such as the liver and spleen, where the LNP concentration peaks after about 8 h (Hassett et al., 2019). Individual cells within the injected tissues take up LNPs through clathrin- and alveolae-mediated endocytosis (Rejman et al., 2005). Although most relevant work has been done on muscle tissue, the same pathways also mediate non-intramuscular delivery such as aerosol delivery to the lungs (Li et al., 2020) and delivery to the eye (Patel et al., 2019).

The efficiency by which individual cells take up LNPs can differ with cell type as well as transcriptional state of the cells (Dobrowolski et al., 2022). The primary destination of injected nanoparticles appear to be muscle cells, various types of immune cells which infiltrate the injection site, and monocytes and other antigen presenting cells in the draining lymph nodes (Liang et al., 2017; Lindsay et al., 2019). Not all cells that take up LNPs also efficiently translate their mRNA cargo, for example, monocytes and dendritic cells produce protein encoded on LNP-delivered transcripts more efficiently than neutrophils despite similar uptake rates (Liang et al., 2017).

Following endocytosis, LNPs and their RNA cargo are routed through the various stages of the endosomal machinery. IVTmRNAs need to escape from the endosome into the cytoplasm to be translated, and this is thought to be one of the most limiting steps of IVTmRNA vaccination strategies (Jiang et al., 2020). In a detailed study of endosomal escape of siRNA-loaded LNPs, escape of endocytosed LNPs was observed within a narrow window around the time of the Rab5-Rab7 conversion step of endosome maturation, 5–15 min after the initiation of endocytic uptake (Wittrup et al., 2015). Endosomal escape strategies are engineered by manipulating LNP composition, for example by including lipids with pKas that interact with the changing pH observed during maturation of endosomal compartments (Hajj et al., 2019; Delehedde et al., 2021). Despite these engineering strategies, typical endosomal escape rates are very low (Deprey et al., 2020; Delehedde et al., 2021), with the great majority of LNPs and their cargo either being degraded, or routed into vesicles which may lead to their re-release into the extracellular medium and subsequent uptake by other cells (Maugeri et al., 2019). Cytoplasmic entry is clearly a limitation in utilising endosomal escape as a mechanism for delivery of IVTmRNAs and almost certainly means much higher amounts of IVTmRNA are required to be administered than if escape were more efficient or alternative routes could be harnessed.

How IVTmRNAs engage with the translational machinery post-endosomal escape is not known in detail, although indirect evidence suggests some possible scenarios. Upon endosomal escape into the cytosol, IVTmRNAs are initially non-protein bound or “naked,” although they may remain associated with some of the LNP components. Cells contain hundreds of unspecific RNA binding proteins (Baltz et al., 2012; Castello et al., 2012), and the crowded intracellular environment (Zimmerman and Minton, 1993) likely drives rapid association of the IVTmRNA with such proteins.

The efficient translation of transfected IVTmRNAs depends on cis-acting sequences, as is the case for cellular transcripts, including 5′- and 3′-UTRs, cap-structure and poly(A) tail (Malone et al., 1989; Jani and Fuchs, 2012). In current IVTmRNA-based therapeutics, the protein-coding ORFs are typically combined with untranslated regions (UTRs) from efficiently translated cellular mRNAs, or with non-natural UTR sequences that have been selected for high translational efficiency (Cao et al., 2021; Fang et al., 2022). The sensitivity of unmodified transcripts to Protein Kinase R (PKR, which is activated by double and single stranded RNA) further suggests that requirements for eIF2 activity (which is the target of PKR) are similar to natural transcripts (Anderson et al., 2010; Cesaro and Michiels, 2021), and the overall time between the introduction of IVTmRNAs into the cell and the emergence of their translation products is similar to transcription-translation delays observed *in vivo* (Reiser et al., 2019). Despite their different route of entry into the cytoplasm, IVTmRNAs are thus most likely translated *via* similar mechanisms as natural cellular transcripts, albeit less efficiently as outlined below.

### 2.3 Consequences of differing entry routes

One of the fundamental differences between natural transcripts and IVTmRNAs is the fact that the latter do not pass through the natural mRNP states that precede engagement of the translational machinery. Any of the processes that have been identified as functionally dependent on the pioneer round of translation, including secretion and antigen presentation, may thus be less efficient for IVTmRNAs.

The synergistic effect from the presence of both a cap structure and the poly(A) tail in IVTmRNAs (Mockey et al., 2006) is similar to that observed in other contexts, where the synergy is known to depend on assembly of a functional cap-binding complex (Borman, 2000; von der Haar et al., 2004). This indicates that IVTmRNAs also require full assembly of this complex. However, unlike for natural transcripts where association with the cytoplasmic cap-binding protein eIF4E has been reported to be catalysed by components of the perinuclear mRNP, IVTmRNAs must somehow associate with non-cap-bound eIF4E encountered in the cytoplasm. Estimates for abundance of eIF4E in human cells vary, but estimates at the lower end (Duncan et al., 1987) are similar to estimates for the total capped transcript content (Marinov et al., 2014), which indicates that available eIF4E may be limiting in actively translating cells. Association between IVTmRNAs and eIF4E may thus limit translational efficiency of the latter. Interestingly, IVTmRNAs were observed to be most efficiently translated when localised near the nucleus (Sayers et al., 2019), the same site that is also the location of first engagement with the translational machinery for most natural transcripts. However, it remains unclear how far perinuclear IVTmRNAs engage with mRNP components that precede the translationally active state.

Multiple lines of evidence indicate that introduction of IVTmRNA-containing LNPs into cells is perceived as a stress that activates various translational control mechanisms. The interaction of nanoparticles with cells can be a cause of stress in itself (Cameron et al., 2022), although the LNPs used for IVTmRNA delivery have been designed to avoid toxic effects (Delehedde et al., 2021). Another cause of cell stress associated with LNP-mediated IVTmRNA delivery are the endosomal rupture events that release LNP cargo into the cytosol, which can result in inflammasome activation, induction of cell death (pyroptosis) of the affected cell, and inflammation in the surrounding tissue (Forster et al., 2022).

In addition to LNP-induced cell stress, the IVTmRNA cargo itself can induce stress pathways. Naked RNA can act as seed material that directly promotes stress granule formation (Bounedjah et al., 2014). Cells transfected with IVTmRNAs frequently show strong activation of PKR (Anderson et al., 2010) which phosphorylates eIF2, thereby ablating global translational activity in transfected cells (Lee et al., 1993). The PKR-dependent loss of translational activity in transfected cells upon introduction of IVTmRNAs can be partially controlled by co-application of inhibitors of the integrated stress response like ISRIB (Ohto et al., 2019) or by using modified uridines in the *in vitro* transcription process (Anderson et al., 2010; Kirschman et al., 2017). The use of modified nucleotides is discussed in more detail in the following section.

## 3 Nucleotide modifications and translational efficiency

Transfection of IVTmRNA frequently results in activation of PKR, which reduces the overall translational efficiency of the transfected cell (see above). Moreover, *in vitro* transcribed RNA stimulates various innate immune receptors, particularly endosomal and cytosolic pattern recognition receptors (PRRs) such as toll-like receptors 7, 8, and 3 that sense single and double stranded RNA, thereby generating its own immune response (Mu and Hur, 2021). Both are undesired activities in the context of RNA therapeutics, and both have been attributed to a self-priming activity associated with the T7 polymerase commonly used for *in vitro* transcription which results in the aberrant production of double stranded RNA (Mu et al., 2018). Although the dsRNA-dependent stimulation of immune receptors appears to be the main component of immune responses against IVTmRNAs, single stranded RNA has also been reported to stimulate immune responses in a RIG-I dependent manner (Pichlmair et al., 2006).

The activation of both PKR and innate immune response pathways can be suppressed by introducing various modified nucleotides into the *in vitro* transcribed RNA (Karikó et al., 2005; Anderson et al., 2010; Durbin et al., 2016). Most current studies agree that the incorporation of such modified nucleotides is necessary to avoid undesired PKR stimulation and immunogenicity, although it is worth noting that some studies reported differing results for particular sequences (Thess et al., 2015; Kauffman et al., 2016). Despite ongoing debate concerning these results, the most commonly administered current IVTmRNA vaccines are transcribed using N1-methyl-pseudouridine instead of uridine (Morais et al., 2021).

**FIGURE 4:**
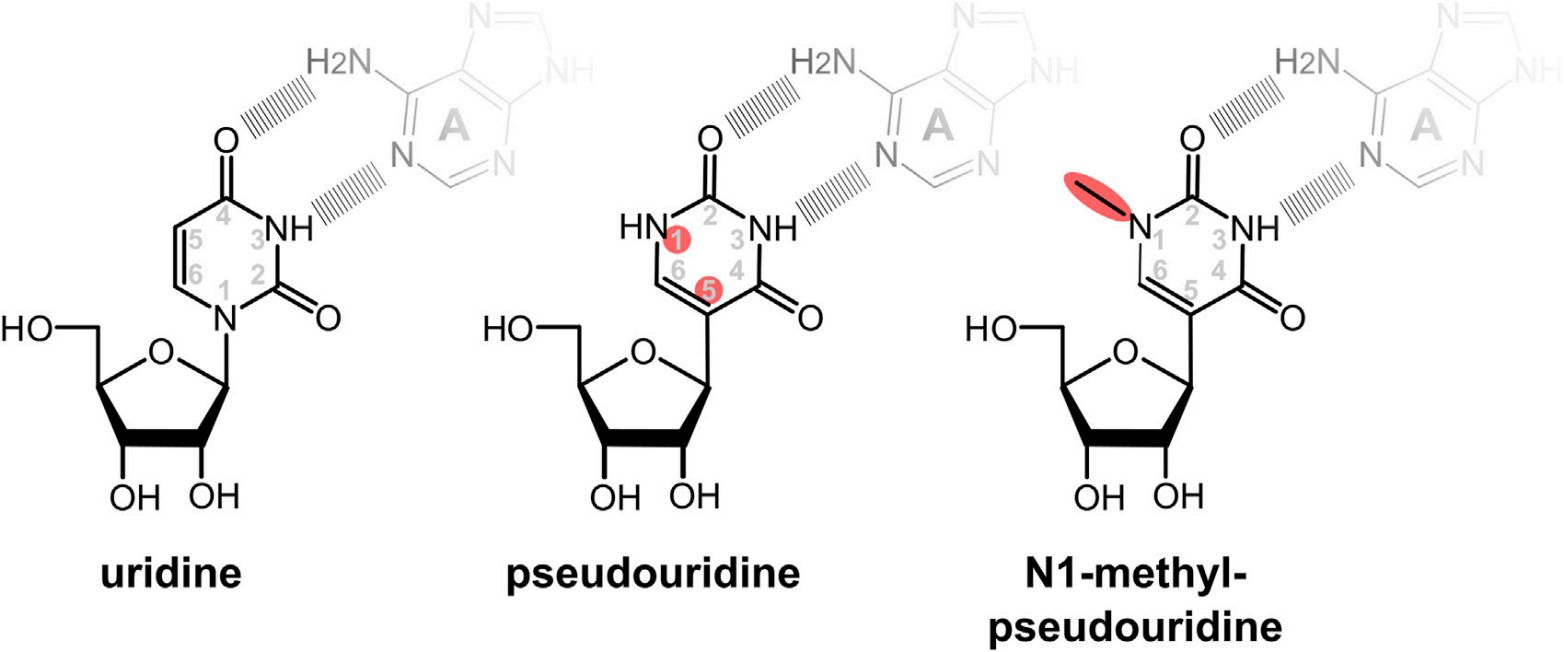
Modified nucleotides used to ablate stimulation of immune responses and PKR activity by IVTmRNAs. In pseudouridine the uracil base is rotated so that the glycosidic bond between the sugar and base is transferred from the N1 to the C5 atom of the base. In the commonly used base N1-methyl-pseudouridine, the N1 atom is attached to an additional methyl group. Despite the rotation of the base, the potential to form base pairing patterns *via* the Watson-Crick face of the uridine is maintained in the modified bases. Sites of changes relative to native uridine are highlighted in red.

Pseudouridine (Ψ) is the most common natural base modification in RNAs, including mRNAs (Schwartz et al., 2014). Although in Ψ the base moiety is rotated relative to the ribose when compared to normal uridine, the orientation of the main hydrogen bond donor and acceptor atoms on the Watson-Crick face are identical in the two bases (Figure 4). Consistently, Ψ can be found base-pairing with adenine in a fashion similar to unmodified uridine in experimental RNA structures (Chawla et al., 2015), as well as forming wobble base pairs with uridine and guanine, again similar to unmodified uridine. Thus, from the point of view of codon decoding and the interaction between Ψ containing codons and their base-pairing anticodons, Ψ is expected to behave similar to uridine.

Despite the similar geometry of their base-pairing hydrogen bonds, experimental studies have shown that U and Ψ form base pairs with other bases with different affinities. In addition to stabilising the canonical base-pair with adenine, Ψ also stabilises wobble-base pairs with guanine and uridine, but not with cytidine (Kierzek et al., 2014). These effects were attributed to changes in the ability of the base-pairs to stack with neighbouring bases, and in the case of the Ψ:A base pair also to a potential additional hydrogen bond involving the N1 atom which is not available for hydrogen bonding in unmodified uridine (Hudson et al., 2013; Kierzek et al., 2014).

Following initial work with pseudouridine, it was found that methylation of its N1 atom (Figure 3) further enhanced the ability of Ψ to suppress activation of immune receptors and the global translation inhibition through activation of PKR (Andries et al., 2015). Presumably for these reasons the predominant current COVID-19 vaccines contain N1-methyl-pseudouridine (m1Ψ) in place of uridine.

It is difficult to compare the translational activity of uridine-, Ψ- and m1Ψ-containing IVTmRNAs directly because effects of the modifications on the transcript itself need to be disentangled from the PKR-dependent global regulation of translation. Using a massively parallel survey of 5′-UTRs, Sample et al. (2019) showed that secondary structures within the UTRs of Ψ and m1Ψ-modified transcripts more strongly inhibit ribosome recruitment compared to natural uridine-containing ones. Svitkin et al. (2017) showed that modified IVTmRNAs also show evidence of stronger ribosomal pausing and decreased translation elongation rates. Thus, on both counts the local effect of the modifications on the transcript is actually inhibitory, and the increased protein yields observed with modified transcripts are most likely due to the overriding effect of alleviating the global inhibition of translation observed with unmodified transcripts.

The inhibitory effect of Ψ and m1Ψ in both UTRs and ORFs may be in part attributable to the effect of the modifications on the stability of secondary structure elements, although increased stability could also result in a prolonged lifetime and therefore prolonged expression of the target antigen. A direct inhibitory effect of Ψ on codon decoding was confirmed *in vitro* with bacterial ribosomes (Eyler et al., 2019). In this study isolated Phe-tRNA^Phe^_GAA_ was observed to show two-fold slower GTPase activation on individual UUU codons in which one or more positions were replaced with Ψ. In crystal structures of *E. coli* ribosomes containing this tRNA and modified codons, placement of the tRNA acceptor stem in the peptidyl transferase centre (PTC) was impaired, indicating that communication between the decoding centre and the PTC is less efficient on Ψ containing codons.

In decoding systems *in vivo*, the overall time required to decode a codon is dependent on the number and type of non-cognate tRNAs that need to be rejected before a cognate tRNA is accepted (Chu et al., 2011; Tarrant and von der Haar, 2014). A particular problem in this respect are near-cognate tRNAs, which can form partial mini-helices between the codon and anti-codon (Plant et al., 2007). Near-cognate tRNAs take much longer to reject than other non-cognates, and carry a non-negligible risk of misincorporation (Plant et al., 2007; Tarrant and von der Haar, 2014). Since Ψ:G and Ψ:U interactions appear to be stabilised compared to U:G and U:U (Kierzek et al., 2014) and both types of interaction occur in some near-cognate tRNAs, Ψ-containing codons can be expected to be more prone to interference from near- and non-cognate tRNAs. Consistent with this expectation, translation elongation rates on fully Ψ-modified transcripts in reconstituted bacterial translation systems were reduced 3-fold and thus greater than expected if the reduced GTPase activation had been the sole contributing factor. Moreover, increased amino acid misincorporation was observed specifically on Ψ-containing codons in bacterial and cultured human cells (Eyler et al., 2019), Ψ incorporation into stop codons was observed to cause increased non-sense suppression (Karijolich and Yu, 2011), and a recent study provided direct evidence for changes in near-cognate tRNA interactions on m1Ψ-modified codons (Monroe et al., 2022). Together these results suggest that modification of transcripts with Ψ or m1Ψ alters competition from near-cognate tRNAs, thereby leading to general ribosome slow-down as well as reduced translational accuracy.

The effects resulting from intended uridine modifications may overlap with effects caused by inadvertent modifications that can occur in IVTmRNAs, notably those arising from the oxidation of nucleotides in RNA. Oxidatively damaged nucleotides in RNA can lead to amino acid misincorporation (Tanaka et al., 2007) and ribosome stalling (Shan et al., 2007). To our knowledge no studies have yet directly assessed levels of oxidative nucleotide damage in RNA vaccines, but oxidation of LNP components such as cholesterol has been shown to occur and may be exacerbated by the presence of impurities in the PEG lipid used in the production of LNP formulations (Uddin et al., 2021).

One consequence of reduced ribosome speed can be ribosome collisions, which cells can perceive as signalling events that elicit a variety of cellular responses. Ribosome collisions can activate Gcn2, p38 and JNK kinases and reduce global translational activity in the affected cell (Wu et al., 2020; Snieckute et al., 2022). At the same time, colliding ribosomes can activate the E3 ubiquitin ligases of the Ribosomal Quality Control pathway (Juszkiewicz et al., 2020) and the ribosomal RNAse activity which is part of the No-Go decay pathway (Doma and Parker, 2006). These surveillance activities jointly promote the degradation of the stalled ribosome:RNA:nascent peptide complex. Svitkin *et al.* observed that membrane association could relieve m1Ψ-dependent ribosome collisions *in vitro* (Svitkin et al., 2022)*,* potentially as a consequence of the altered translational dynamics of membrane associated ribosomes. In sum, whether ribosome collisions occur on modified or unmodified therapeutic mRNAs *in vivo* is unknown, although if collisions do occur they are likely to reduce the efficiency with which such transcripts are translated.

How much protein an mRNA can produce can be limited either by the translation initiation rate or by the translation elongation rate, depending on the ratio of the two activities (Chu et al., 2014; Tarrant and von der Haar, 2014). While the majority of cellular mRNAs is likely limited by initiation rates, regulatory regions of many recombinant protein encoding constructs are derived from highly translated natural transcripts and such constructs are therefore frequently limited by their elongation rates unless the codon usage of the transcript has been specifically optimised (Chu et al., 2014). If IVTmRNAs containing modified uridines are decoded more slowly than natural transcripts, elongation rates are likely more limiting, potentially increasing the importance of codon usage optimisation for efficient protein expression. The RNA vaccines currently in clinical use show clear signs of codon optimisation as they avoid the use of codons that are infrequently used in humans (Figure 4). Although some analyses of the codon usage in these vaccines have been published (Xia 2021) the exact optimisation algorithms used to generate these sequences have not been disclosed.

**FIGURE 5:**
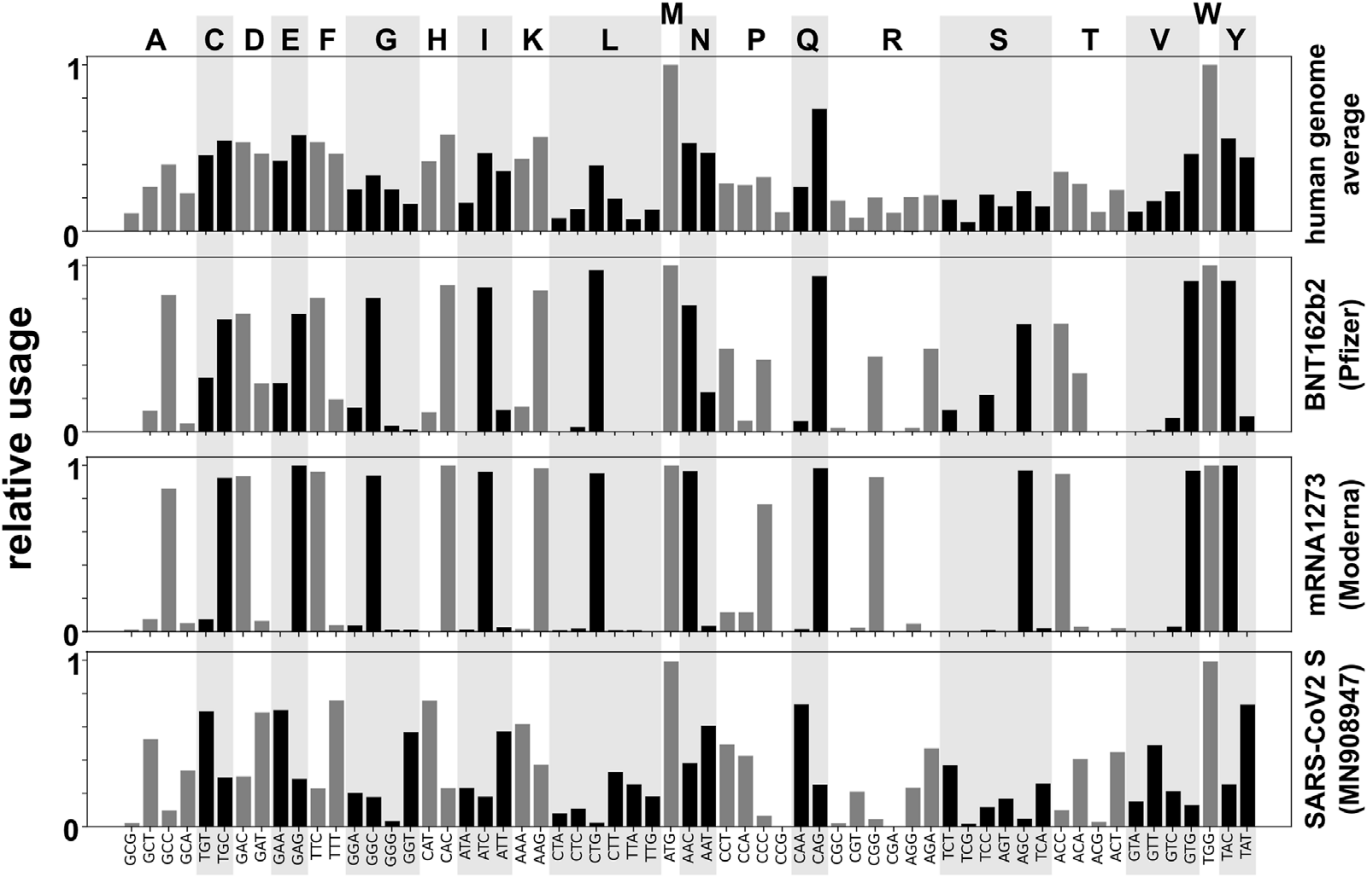
Codon usage for sense codons in two clinically used SARS-CoV-2 vaccines, compared to the average codon usage in open reading frames in the human genome (top) and to codon usage in a representative native spike protein encoding sequence from SARS-CoV-2 (bottom, from Genbank ID MN908947).

Different studies have emphasized various design principles for nucleic acid-based vaccines. These include copying codon usage from highly expressed mammalian genes, which improves translational dynamics and also increases the GC content of the ORF (André et al., 1998); more fine-grained design considerations that allow controlling protein folding and posttranslational modifications which may be adversely affected by “over-optimising” codon usage (Katneni et al., 2022), strategies that avoid PKR activation and immune-stimulation without the use of modified nucleotides (Thess et al., 2015), and strategies that emphasize the control of a transcript’s half-life by controlling secondary structure motifs (Geisberg et al., 2014). While the currently used vaccines were clearly designed with some reference to human codon usage frequencies this has not been implemented rigorously, with both the Pfizer and Moderna vaccines containing some sub-optimal codons for most amino acids (Figure 5). These non-optimal codon choices may have been introduced due to functional considerations such as those outlined above, although to or knowledge information on what such considerations may have entailed has not been made public.

## 4 Summary

Although IVTmRNAs are translated by the cellular gene expression machinery, their genesis and route of entry into the cell are fundamentally different from native transcripts. In consequence the location of their first engagement with the translational machinery, their arrival as a non-protein bound, “naked” RNA molecule rather than a pre-formed mRNP, and the presence of modified nucleotides in high densities all have consequences for the efficiency with which IVTmRNAs are translated. Understanding these differences in better detail is required if protein synthesis rates of therapeutic mRNAs are to be improved.

Although therapeutics currently in clinical use are clearly efficient enough to form effective vaccines, the expression levels required to stimulate immune responses are relatively low. The various modes for non-vaccine therapeutics that are currently being explored will require higher expression levels, and for such applications removing the translational limitations of current constructs is of key importance.

## Footnotes

1 See for example the European Medicines Agency assessment reports for Comirnaty (https://www.ema.europa.eu/en/documents/assessment-report/comirnaty-epar-public-assessment-report_en.pdf) and for Spikevax (https://www.ema.europa.eu/en/documents/assessment-report/spikevax-previously-covid-19-vaccine-moderna-epar-public-assessment-report_en.pdf).
